# The Impact of Hospital Transfers on Surgical Delay and Associated Postoperative Outcomes for Hip Fracture Patients in Scotland: A Cohort Study

**DOI:** 10.3390/jcm13092546

**Published:** 2024-04-26

**Authors:** Liam Lennox, Phyo K. Myint, Santosh Baliga, Luke Farrow

**Affiliations:** 1 Institute of Applied Health Sciences, University of Aberdeen, Aberdeen AB25 2ZN, UK; 2 Grampian Orthopaedics, Aberdeen Royal Infirmary, Aberdeen AB25 2ZN, UK

**Keywords:** Hip fracture, Scotland, outcomes, delay, transfer, rural, SHFA

## Abstract

**Background/Objectives**: Hip fractures exert a substantial burden on hospital systems. Within Scotland 20% of the population resides rurally, warranting investigation of how this impacts prompt access to surgical care. This study aims to determine whether indirect hospital admission via hospital transfer affects the likelihood of surgical management within 36 h for hip fracture patients. **Methods**: A retrospective cohort study was performed. This used Scottish Hip Fracture Audit data including patients aged ≥50 split into two propensity matched groups based on their transfer status. Descriptive analysis compared patient characteristics. Regression assessed achieving surgery within 36 h of admission in the unmatched and matched cohorts. Secondary outcomes included time to surgery, mortality, mobilization, returning to residence and length of stay. A sensitivity analysis was undertaken to assess for residual confounding effects. **Results**: The unmatched analysis included 20,132 patients. Transfer patients were younger (*p* = 0.007) and less-comorbid (*p* < 0.001). In the matched population, 711 (63.6%) transfer patients had surgery with 36 h of presentation to hospital, compared to 852 (75.3%) non-transfer patients. Transfer patients had 43% reduced odds of timely surgery (OR (95% CI) 0.57 (0.48 to 0.69); *p* < 0.001). No disparities emerged in mortality, mobilisation or returning to residence., Transfer patients experienced a significant increase in length of stay in hospital (median (IQR) 16 (8 to 33) vs. 13 (8 to 30); *p* = 0.024). **Conclusions**: Hospital transfer is associated with significantly reduced odds of timely surgery, a longer time to surgery and longer length of stay. Development of structured network pathways that minimize delay to transfer are required to potentially optimize outcomes and reduce associated cost.

## 1. Introduction

Hip fractures pose significant challenges to healthcare systems worldwide, including in Scotland where around 7000 patients require hospital admission annually [1]. The annual direct costs of hip fracture admissions in the UK exceeds £2 billion, with further financial consequences due to lost productivity from morbidity and mortality [2]. Scotland, like many advanced economies, is experiencing an ageing population due to a longer life expectancy and reduced birth rate [3]. Consequently, it is anticipated that the number of hip fracture cases will increase, imposing a greater burden on the NHS and affected individuals [4]. Surgical treatment is the primary approach for most hip fracture cases [5]. Notable, 20% of Scotland’s population resides in rural communities, making it essential to investigate potential associations between transfer status and delays in surgical management [6].

The Scottish Standards of Care for Hip Fracture Patients (SSCHFP) was developed to reduce variation in hip fracture care across Scotland, whilst further enhancing the quality of clinical care [7]. Previous research has demonstrated adherence to standard six (surgery within 36 h of admission)—is associated with improved patient outcomes [8]. Furthermore, delayed surgery has previously been associated with adverse postoperative outcomes in large meta-analysis, including mortality rates, complications, and extended hospital stays [9,10]. While limited international studies have explored the associated between hospital transfer and delay in surgical management [11,12,13,14,15,16], none however have addressed the unique geographical challenges faced by Scotland and the associated large rural population, particularly in the Highlands and Islands. This means that several patients do not have direct access to hospitals with hip fracture services, and instead initially present to small local units designed to manage rehabilitation or minor injuries.

The authors hypothesis that hospital transfer in Scotland may be associated with delays in receiving surgical management within 36 h for hip fracture patients. This study aimed to analyse the Scottish Hip Fracture Audit (SHFA) to determine if indirect admission via hospital transfer impacts the likelihood of surgical management within 36 h of admission for hip fracture patients aged 50 and over in Scotland. Secondary aims explored associations between transfer status and other patient outcomes based on SSCHFP guidelines [7].

## 2. Materials and Methods

### 2.1. Study Design, Setting, and Participants

A retrospective analysis of cohort data was undertaken using anonymised audit data prospectively collected by the SHFA between January 2019 and December 2021. The chosen period reflects when detailed information about transferred patients was available. Data was collected from all trauma centers in Scotland, and local audit coordinators ensured data quality and robustness [17]. This study included all patients over the age of 50 in Scotland who experienced an acute hip fracture between January 2019 and December 2021. Patients managed conservatively, those with extensive trauma, a known pathological fracture or who suffered an in-hospital fall were excluded.

### 2.2. Data Collection

Anonymised data was obtained from the SHFA database through Public Health Scotland (PHS). The primary explanatory variable of interest was the patient’s transfer status (transfer/non-transfer), Other demographic and patient variables were age, sex, residence prior to admission, American Society of Anaesthesiologists (ASA) grade, operation type, 4AT score and Scottish Index of multiple deprivation decile (SIMD) [18]. The primary outcome of interest was receiving surgery within 36 h of admission. Secondary outcomes included time to surgery, mortality at 30 and 60 days postoperatively, returning to original residence by day 30 postoperatively, mobilisation by day one postoperatively, total length of stay (LOS), and acute LOS [7]. LOS was truncated at 60 days.

### 2.3. Sample Size

An a priori sample size calculation was conducted which indicated a maximum of 1380 patients (690 per group) were required to detect a 10% difference in the odds of achieving surgery within 36 h of admission between the groups at 80% power and *p* < 0.05. The SHFA contained 20,430 non-transfer and 1213 transfer patients potentially eligible for inclusion between January 2019 and December 2021.

### 2.4. Statistical Analysis

Analysts had access to an anonymised dataset containing the requested variables obtained from PHS. Initial data visualization was performed to assess the data characteristics. Data cleaning was undertaken, and missing values were recoded using the SHFA data dictionary [19]. All variables had less than 3.5% missing data, except for 4AT score (21.9%) and ASA grade (17.8%; Appendix A). It was confirmed that all missing data were missing at random or missing completely at random. The multiple imputation of chained equation random forest algorithm (MICE) was used to impute missing data for all explanatory fields. All outcome variables had <1% missing data and pairwise deletion was used to manage this.

The study population was dichotomised into two groups based on their transfer status. Time to surgery was calculated by subtracting the date and time of admission from the date and time of surgery. 4AT scores were categorised based on the rapid clinical test for delirium interpretation [20]. Descriptive analysis was performed to examine differences in patient variables by transfer status. Visualisation of histograms and Shapiro-Wilks tests confirmed continues variables to be non-normally distributed, thus they were presented as medians with their interquartile range (IQR). Categorical variables were reported as a number with percentages. Pearson’s chi-squared tests were employed to assess differences in the predictor variables between the two groups. Chi-squared tests with continuity correct were used when categorical variables contained two groups.

To address heavy imbalances in group sizes, the non-transfer group was matched to the transfer group using nearest neighbour propensity score matching with a one-to-one ratio by all explanatory variables [21]. Analysis of outcome variables was performed in both the unmatched and matched populations. The association between the transfer status and dichotomous outcome variables was assessed using unadjusted logistic regression. Mann-Whitney U tests were used to assess the association between transfer status and time to surgery, acute LOS and total LOS. A sub-group analysis stratifying patients transferred from islands was also undertaken, where Kruskal-Wallis tests were used to identify differences in continuous outcomes. To explore the potential effects of unmeasured confounders relating to patient frailty, we conducted a sensitivity analysis which replicated the main analysis but for patients aged 80 and over who were not admitted from home, representing the frailest individuals in the study population. All statistical analysis was performed using R (version 4.2.0). Statistical significance was determined by *p* < 0.05.

### 2.5. Ethics

The service evaluation nature of this project and the use of anonymised secondary data meant ethical approval was not required. Subsequent PHS approval was granted in May 2023 (DP23240035). This study was conducted in accordance with the Declaration of Helsinki [22], and the Caldicott principles regulating the use of patient data [23]. This study was reported in accordance with the REporting of studies Conducted using Observational Routinely collected health data (RECORD) statement [24].

## 3. Results

### 3.1. Participants

**Figure 1:**
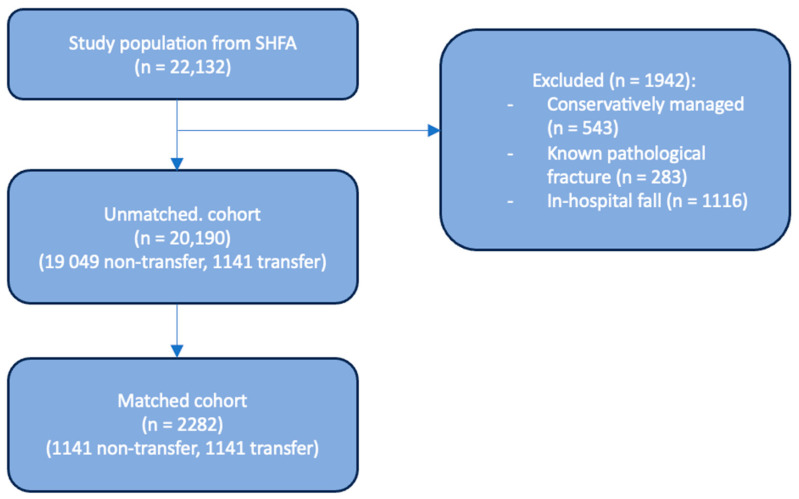
Flowchart showing patient selection for the sample population.

Initially, 22,132 patients were included from the SHFA datasbase within the specified time frame. Following exclusion criteria there were 20,190 participants, with 19,049 in the non-transfer group and 1141 in the transfer group (Figure 1). Table 1 reports the patient characteristics of these two groups, Appendix A Table A1 describes characteristics prior to data imputation.

### 3.2. Unmatched Study Population

A total of 20,190 participants were included in the descriptive analysis for the unmatched population. The two groups demonstrated significant differences in all explanatory variables, except for sex (males: 30.0% vs. 29.4%; *p* = 0.712). Transferred patients were notably younger (<80 years: 45.6% vs. 39.7%; *p* = 0.007) and predominantly from middle-class socio-economic backgrounds (SIMD 4 to 7: 52.7% vs. 40.1%; *p* < 0.001). Additionally, more transferred patients were admitted from home (85.0% vs. 80.7%; *p* < 0.001), had a lower frequency of delirium (18.9% vs. 23.5%; *p* < 0.001) and had a better overall health status (ASA < 3: 30.1% vs. 25.3%; *p* < 0.001).

Table 2 reports the clinical outcomes for the unmatched groups. The median (IQR) time to surgery (hours) was found to be significantly greater in the transfer patients than non-transfer patients (30.1 (18.2 to 41.1) vs. 21.2 (15.3 to 35.7); *p* < 0.001). Among the transfer patients 711 (63.6%) underwent surgery within 36 h of admission, in contrast to 14,320 (75.6%) non-transfer patients (OR (95% CI) 0.56 (0.50 to 0.64); *p* < 0.001).

Transfer patients were significantly found to have 28% reduced odds of 30-day mortality (OR (95% CI 0.72 (0.54 to 0.94); *p* = 0.022) and 29% reduced odds of 60-day mortality (OR (95%) 0.71 (0.57 to 0.88); *p* = 0.003). Transfer patients were also significantly found to have 16% increased odds of returning to their original residence within 30-days postoperatively (OR (95% CI) 1.16 (1.03 to 1.32); *p* = 0.017) and 27% increased odds of mobilising by day one postoperatively (OR (95% CI) 1.27 (1.11 to 1.46); *p* < 0.001). There was found to be no difference in both the acute and total LOS between the groups.

#### 3.2.1. Matched Study Population

**Figure 2:**
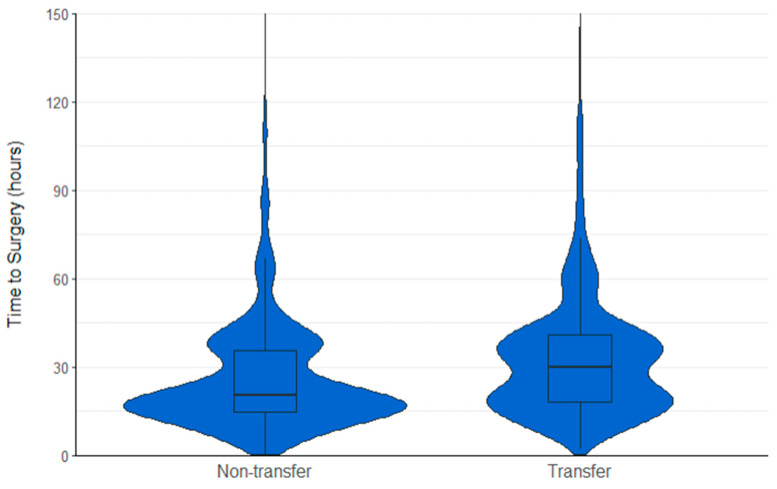
Violin plot showing time to surgery (hours) for non-transfer and transfer patients, outliers truncated at 150 h.

**Figure 3:**
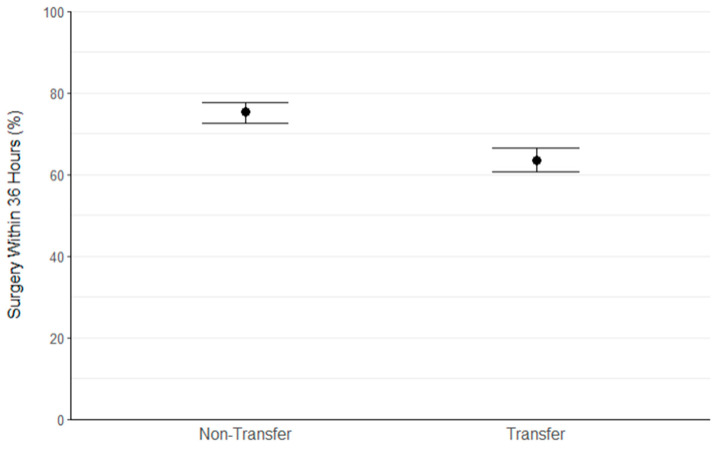
Error bar chart showing the percentage of patients within the non-transfer and transfer groups to achieve surgery within 36 h of admission.

Following the propensity matching procedure, 2084 participants were retained (1141 transfer, 1141 non-transfer). Standardised differences for all baseline characteristics were less than 3.5%, indicating the. Successful balancing of groups (Table 1). Table 3 presents the association between transfer status and clinical outcomes in the matched population. The median (IQR) time to surgery (hours) for transfer patients was found to be significantly greater (30.1 (18.2 to 41.1) vs. 20.3 (14.5 to 35.9); *p* < 0.001); Figure 2). Among the transfer patients, 711 (63.6%) underwent surgery within 36 h of admission, compared to 852 (75.3%) for non-transfer patients (Figure 3). Transfer patients were found to have 43% reduced odds of achieving surgery within 36 h of admission (OR (95% CI) 0.57 (0.48 to 0.69); *p* < 0.001).

**Figure 4:**
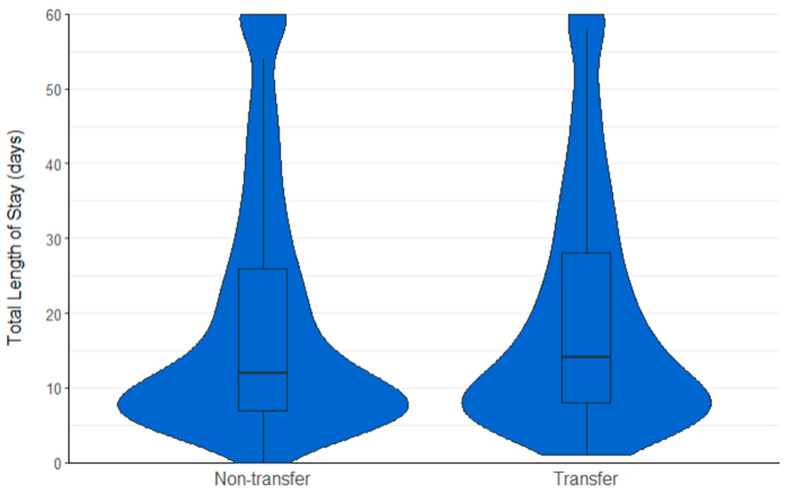
Violin plot showing the total LOS (days) for non-transfer and transfer patients.

There was found to be no significant difference between transfer patients for 30-day mortality (4.9% vs. 5.3%; *p* = 0.714), 60-day mortality (8.1% vs. 9.4%; *p* = 0.284), return to residence postoperatively (63.7% vs. 65.8%; *p* = 0.293), mobilisation postoperatively (75.3% vs. 74.7%; *p* = 0.735) and acute LOS (median (IQR) 9 (6 to 14.5) vs. 9 (6 to 13) days; *p* = 0.064). Transfer patients were however significantly found to experience three extra days in hospital (16 (8 to 33) vs. 13 (8 to 30) days; 0.024); Figure 4).

#### 3.2.2. Matched Study Population Island Sub-Group

Within the 1141 transfer patients, 142 (12.4%) were transferred from islands (Table 4). The median (IQR) time to surgery (hours) for transferred island patients was found to be singificantly greater than non-transfer patients (39.7 (29.0 to 58.5) vs. 20.3 (14.5 to 35.9); *p* < 0.001). Among the transferred island patients, 56 (40.6%) underwent surgery within 36 h of admission, compared to 655 (66.8%) patients transferred from the mainland. Transferred island patients were significantly found to have 78% reduced odds of achieving surgery within 36 h of admission compared to non-transfer patients (OR (95% CI) 0.22 (0.16 to 0.32); *p* < 0.001). Transferred island patients were also found to experience a longer acute LOS than both non-transfer (median (IQR) 10 (8 to 15.8) vs. 9 (6 to 13) days; *p* = 0.005) and mainland transferred patients (median (IQR) 10 (8 to 15.8) vs. 9 (6 to 14) days; *p* < 0.001). Transferred island patinets also experienced significantly longer total LOS than both non-transfer (median (IQR) 23 (12 to 37) vs. 13 (8 to 30) days; *p* < 0.001) and mainland transferred patients (median (IQR) 23 (12 to 37) vs. 15 (8 to 32) days; *p* < 0.001).

### 3.3. Sensitivity Analysis

Table 5 reports postoperative outcomes for participants aged 80 and over not admitted from home, representing the frailest patients in the population. This included 274 participants (137 in the transfer group and 137 in the non-transfer group). Also in this population, transfer patients experienced significantly longer times to surgery (median (IQR) 26.4 (17.5 to 38.2) vs. 19.4 (14.2 to 28.8) hours; *p* < 0.001). Transfer patients had further significantly reduced odds of receiving surgery within 36 h of admission, experiencing 58% reduced odds (OR (95%CI) 0.42 (0.23 to 0.74); *p* = 0.003).

In this sub-group unlike previously, transfer patients had 59% increased odds of suffering mortality within 30 days (OR (95%) 1.59 (0.78 to 3.34); *p* = 0.208) and 14% increased odds within 60 days (OR (95% CI) 1.14 (0.65 to 2.03); *p* = 0.640). These differences were not however statistically significant. Transfer patients were also found to have reduced odds of returning to their original residence (OR (95% CI) 0.74 (0.44 to 1.27); *p* = 0.821) and achieving early postoperative mobilisation (OR (95% CI) 0.78 (0.49 to 1.27); *p* = 0.332), but this was also not statistically signiicant.

## 4. Discussion

This study aimed to determine if indirect hospital admission via hospital transfer impacts the likelihood of surgical management within 36 h of admission for hip fracture patients aged 50 and over in Scotland. We found that transferred patients experienced longer times to surgery and were significantly less likely to undergo surgery within 36 h of admission. This finding aligns with our initial hypothesis and suggests that hospital transfer may be associated with delays in surgical management. This study did not however find any differences for secondary outcomes such as mortality, return to residence, postoperative mobilisation and acute LOS. We did however reveal that transferred patients experienced a longer total LOS. Transferred patients were also more likely to be younger and healthier than non-transferred patients. When patients were transferred from islands as opposed to rural hospitals on the mainland they experienced further delays to their management and more time in hospital.

Our findings agree with the previous evidence exploring the relationship between transfer status and delays to surgery [11,13,14,15,16], except for one Irish study which found no differences in the odds of achieving timely surgery between the two groups [12]. This could be because Ireland’s geography and healthcare system varies from Scotland’s, and they utilised a target time of 48 h. The study was also conducted ten years ago, when demands on orthopaedic services were less and patients were not as complicated [25]. Considering both our findings and the existing evidence base, there are strong suggestions that transfer status is associated with delays to surgery.

These delays might have been unavoidable, such as needing to optimise a patient’s health status or long-term medication prior to surgery [26]. However, many of these delays are likely avoidable, being the consequence of inefficient referral pathways, limited capacity of operative rooms or availability of surgical personnel [27]. There is evidence however that delays are appropriate to optimise the medical condition of certain patient groups [28,29,30].

Our findings suggest that, despite experiencing delays to surgery, transfer patients experience the same postoperative outcomes as non-transfer patients. This conflicts many large systematic reviews and meta-analysis that have demonstrated associations between delayed surgery and adverse outcomes [9,10,31]. Strong associations between frailty and adverse outcomes have been demonstrated in the past following surgical treatment for hip fractures [32,33,34,35]. Despite not being statistically significant, likely because of inadequate power creating imprecision, our sensitivity analysis did demonstrate a trend towards greater mortality and reduced odds of mobilisation and returning to residence for transfer patients when investigating the frailest patients in our sample. This suggests we did not discover any differences in postoperative outcomes possibly because of residual confounding relating to patient frailty existing in the main analysis, despite efforts to control this. Similar to major trauma patients it is possible that those with a delay to theatre related to hospital transfer exhibit a “second hit” phenomenon, which has not been adequately investigated in this population to date.

The characteristics of our sample population suggests transfer patients are younger and less co-morbid than non-transfer patients. This conflicts recent data from the Scottish government and a study undertaken by Teckle et al. which reveals Scotland’s rural population is older and has more co-morbidities than those living in cities [36,37]. Despite this, they did identify that more people in rural communities live at home, suggesting a lower prevalence of frailty. Consequently, it is unclear if our transfer cohort is healthier than the rural population, they represent because of hospitals selectively referring healthier patients for more complicated procedures such as a Total Hip Replacement.

We identified a significantly longer total LOS for transferred patients, which is consistent with the existing literature [38,39,40]. Limited rehabilitation resources in rural Scotland could require that transfer patients are rehabilitated further prior to discharge, or discharge planning procedures could be more complicated. In 2017, the cost of each excess bed day in hospital was £351 [41]. Therefore, when adjusted for inflation, delays discharging transferred patients costs at least £1,528,347 per year in 2024. More importantly, additional consequences include lost productivity, a worse patient experience, deterioration of general health, additional stress for staff and reduced availability of beds [42].

The main strengths of this study lie in its use of the large and comprehensive dataset collected by the SHFA [17]. This facilitated a substantial sample size, excellent data quality and a nationally representative cohort reflective of hip fracture care within a developed healthcare system. To our knowledge, this study is the first to investigate the association between transfer status and delays in surgical management for hip fractures in Scotland and contributes to the small international evidence base [11,12,13,14,15,16].

Performing imputation by MICE better accounts for statistical uncertainty than other methods and considers the relationships that exist between variables [43,44]. Propensity matching better accounts for unseen variables causing differences between the two groups than adjusted regression, whilst addressing the imbalances in group sizes [21,45,46]. However, this did cause a loss of data and could limit the generalisability of findings. Preserving transfer cases with uncommon characteristics using nearest neighbour matching instead of coarsened exact matching reduced this risk.

A key limitation of our study was using secondary, aggregated data as we were unable to collect all potential confounding variables, which likely caused residual confounding effects in our analysis. Hip fracture care is complicated and influenced by numerous factors which this study could not account for. In the past, ASA grade has demonstrated validity as an indicator of preoperative health status [47]. Previous research identified the availability of ortho-geriatric services to significantly impact patient outcomes [48,49,50]. Considering more than 50% of our population was over 80 years old, this would be an important factor to control.

We were not able to describe and control for where patients have been transferred from and why they were transferred. Some rural practices selectively refer healthier patients for more complicated procedures as opposed to transferring all cases. This could have created a healthier transfer population with residual confounding.

Utilising multi-center national data allows this study to be generalised to all of Scotland. Considering our findings agree with international literature, the findings perhaps could be inferred to other countries with similar geographical and population characteristics as Scotland. Since less than 5% of the population was younger than 60, care should be taken inferring results to younger patients.

Future research should address the limitations of this study to attempt to more definitively determine if the delay experienced by transferred patients is associated with worse postoperative outcomes. Residual confounding must be addressed by considering all important confounding variables. A qualitative aspect exploring healthcare professionals’ beliefs regarding obstructions to achieving time targets for surgery and discharging patients would provide valuable information. Long term outcomes such as 1-year mortality should also be explored, as well as patient reported outcomes including pain, quality of life and functionality. A comprehensive economic analysis would be required for any future policy changes.

## 5. Conclusions

In conclusion, hospital transfer is significantly associated with reduced odds of achieving surgical management for hip fractures within 36 h of admission, a longer time to surgery, and a greater total LOS in Scotland. Despite this, transfer patients do not experience worse postoperative outcomes. It is unclear however if this is the result of residual confounding effects. Future research is required to address the limitations of this study to determine if hospital transfer is associated with worse postoperative outcomes in Scotland.

## Figures and Tables

**Table 1:** Characteristics of included hip fracture patients and associations with transfer status.

	Unmatched				Matched			
	Non-Transfer (n = 19,049)	Transfer (n = 1141)	Std Diff, %	*p*-Value	Non- Transfer (n = 1141)	Transfer (n = 1141)	Std Diff, %	*p*-Value
Sex, n (%)								
Female	13,446 (70.6)	799 (70.0)	−1.2	0.712 ^a^	805 (70.6)	799 (70.0)	−1.2	0.819 ^a^
Male	5603 (29.4)	342 (30.0)	1.2		336 (29.4)	342 (30.0)	1.2	
Age, n (%)								
50 to 54	300 (1.6)	30 (2.6)	6.6	0.003 ^b^	28 (2.5)	30 (2.6)	1.1	0.999 ^b^
55 to 59	519 (2.7)	38 (3.3)	3.4		38 (3.3)	38 (3.3)	0.0	
60 to 64	752 (4.0)	54 (4.7)	3.7		46 (4.0)	54 (4.7)	3.3	
65 to 69	1139 (6.0)	73 (6.4)	1.7		77 (6.7)	73 (6.4)	−1.4	
70 to 74	2057 (10.8)	128 (11.2)	1.3		129 (11.3)	128 (11.2)	−0.3	
75 to 79	2779 (14.6)	198 (17.4)	7.3		198 (17.4)	198 (17.4)	0.0	
80 to 84	3947 (20.7)	228 (20.0)	−1.9		223 (19.5)	228 (20.0)	1.1	
85 to 89	4207 (22.1)	221 (19.4)	−6.9		230 (20.2)	221 (19.4)	−2.0	
90 to 94	2525 (13.3)	125 (11.0)	−7.4		126 (11.0)	125 (11.0)	−0.3	
95+	824 (4.3)	46 (4.0)	−1.5		46 (4.0)	46 (4.0)	0.0	
SIMD, n (%)								
1	1897 (10.0)	72 (6.3)	−15.0	<0.001 ^b^	79 (6.9)	72 (6.3)	−2.5	1.000 ^b^
2	2024 (10.6)	105 (9.2)	−4.9		109 (9.6)	105 (9.2)	−1.2	
3	2058 (10.8)	118 (10.3)	−1.5		114 (10.0)	113 (9.9)	1.2	
4	1883 (9.9)	113 (9.9)	0.1		114 (10.0)	113 (9.9)	−0.3	
5	1954 (10.3)	185 (16.2)	16.2		189 (16.6)	185 (16.2)	−1.0	
6	1914 (10.0)	177 (15.5)	15.1		178 (15.6)	177 (15.5)	−0.2	
7	1883 (9.9)	127 (11.1)	4.0		120 (10.5)	127 (11.1)	2.0	
8	1760 (9.2)	97 (8.5)	−2.7		95 (8.3)	97 (8.5)	0.6	
9	1893 (9.9)	95 (8.3)	−5.8		91 (8.0)	95 (8.3)	1.3	
10	1783 (9.4)	52 (4.6)	−23.0		52 (4.6)	52 (4.6)	0.0	
Residence prior to admission, n (%)								
Home	15,372 (80.7)	970 (85.0)	12.1	<0.001 ^a^	982 (86.1)	970 (85.0)	−3.0	0.513 ^a^
Not home	3677 (19.3)	171 (15.0)	−12.1		159 (13.9)	171 (15.0)	3.0	
4AT, n (%)								
Delirium unlikely	9625 (50.5)	669 (58.6)	16.5	<0.001 ^b^	678 (59.4)	669 (58.6)	−1.6	0.928 ^b^
Possible cognitive impairment	4947 (26.0)	256 (22.4)	−8.5		252 (22.1)	256 (22.4)	0.8	
Possible delirium	4477 (23.5)	216 (18.9)	−11.7		211 (18.5)	216 (18.9)	1.1	
ASA, n (%)								
1	360 (1.9)	34 (3)	6.4	<0.001 ^b^	37 (3.2)	34 (3.0)	−1.5	0.902 ^b^
2	4462 (23.4)	309 (27.1)	8.2		320 (28.1)	309 (27.1)	−2.2	
3	11,357 (59.6)	608 (53.3)	−12.7		607 (53.2)	608 (53.3)	0.2	
4 and 5	2870 (15.1)	188 (16.5)	4.0		177 (15.5)	190 (16.7)	2.8	
Operation type, n (%)								
Fixation	8631 (45.3)	439 (38.5)	−14.1	<0.001 ^b^	432 (37.9)	439 (38.5)	1.3	0.945 ^b^
Hemi arthroplasty	9170 (48.1)	606 (53.1)	10.0		614 (53.8)	606 (53.1)	−1.4	
THR	1248 (6.6)	96 (8.4)	6.7		95 (8.3)	96 (8.4)	0.3	

Std diff, standard difference; ASA, American Society of Anaesthesiologists physical status classification system; 4AT, rapid clinical test for delirium detection; THR, total hip replacement; n, number; %, percent. ^a^ Chi-squared test with continuity correction. ^b^ Pearson’s chi-squared test.

**Table 2:** Postoperative outcomes in the unmatched study population.

	Non-Transfer (n = 19,049)	Transfer (n = 1141)	OR (95% CI)	*p*-Value
Surgery within 36 h, n (%)	14 320 (75.6)	711 (63.6)	0.56 (0.50 to 0.64)	<0.001 ^a^
Time to surgery (hours), median (IQR)	21.2 (15.3 to 35.7)	30.1 (18.2 to 41.1)	-	<0.001 ^b^
30-day mortality, n (%)	1270 (6.7)	56 (5.2)	0.72 (0.54 to 0.94)	0.022 ^a^
60-day mortality, n (%)	2079 (11.0)	90 (9.1)	0.71 (0.57 to 0.88)	0.003 ^a^
Return to residence, n (%)	11 459 (60.2)	727 (63.7)	1.16 (1.03 to 1.32)	0.017 ^a^
Postoperative mobilisation, n (%)	13 450 (70.6)	859 (75.3)	1.27 (1.11 to 1.46)	<0.001 ^a^
Acute LOS (days), median (IQR)	9 (6 to 14)	9 (6 to 14.5)	-	0.553 ^b^
Total LOS (days), median (IQR)	16 (8 to 35)	16 (8 to 33)	-	0.706 ^b^

n, number; %, percent; IQR, interquartile range; LOS, length of stay; OR, odds ratio; CI, confidence interval. ^a^ Unadjusted logistic regression. ^b^ Mann-Whitney U test.

**Table 3:** Postoperative outcomes in the matched study population.

	Non-Transfer (n = 1141)	Transfer (n = 1141)	OR (95% CI)	*p*-Value
Surgery within 36 h, n (%)	852 (75.3)	711 (63.6)	0.57 (0.48 to 0.69)	<0.001 ^a^
Time to surgery (hours), median (IQR)	20.3 (14.5 to 35.9)	30.1 (18.2 to 41.1)	-	<0.001 ^b^
30-day mortality, n (%)	60 (5.3)	56 (4.9)	0.93 (0.64 to 1.36)	0.714 ^a^
60-day mortality, n (%)	106 (9.4)	90 (8.1)	0.85 (0.63 to 1.14)	0.284 ^a^
Return to residence, n (%)	751 (65.8)	727 (63.7)	0.91 (0.77 to 1.08)	0.293 ^a^
Postoperative mobilisation, n (%)	852 (74.7)	859 (75.3)	1.03 (0.85 to 1.25)	0.735 ^a^
Acute LOS (days), median (IQR)	9 (6 to 13)	9 (6 to 14.5)	-	0.064 ^b^
Total LOS (days), median (IQR)	13 (8 to 30)	16 (8 to 33)	-	0.024 ^b^

n, number; %, percent; IQR, interquartile range; LOS, length of stay; OR, odds ratio; CI, confidence interval. ^a^ Unadjusted logistic regression. ^b^ Mann-Whitney U test.

**Table 4:** Post-operative outcomes including island sub-group.

	Non-Transfer (n = 1141)	Transfer (Mainland) (n = 999)	Transfer (Island) (n = 142)	OR (95% CI)	*p*-Value
Surgery within 36 h, n (%)	852 (75.3)	655 (66.8)	56 (40.6)	Mainland = 0.42 (0.23 to 0.74) Island = 0.22 (0.16 to 0.32)	Mainland <0.001 ^a^ Island <0.001 ^a^
Time to surgery (hours), median (IQR)	20.3 (14.5 to 35.9)	28.1 (17.5 to 39.9)	39.7 (29.0 to 58.5)	Mainland = 5.6 (3.2 to 8.0) Island = 21.2 (16.1 to 26.2)	Mainland <0.001 ^b^ Island <0.001 ^b^
30-day mortality, n (%)	60 (5.3)	52 (5.2)	4 (2.9)	Mainland = 0.99 (0.68 to 1.45) Island = 0.52 (0.16 to 1.30)	Mainland = 0.967 ^a^ Island = 0.217 ^a^
60-day mortality, n (%)	106 (9.4)	80 (9.8)	10 (8.1)	Mainland = 0.86 (0.64 to 1.17) Island = 0.76 (0.36 to 1.41)	Mainland = 0.351 ^a^ Island = 0.415 ^a^
Return to residence, n (%)	751 (65.8)	640 (64.1)	87 (61.3)	Mainland = 0.93 (0.77 to 1.11) Island = 0.82 (0.58 to 1.18)	Mainland = 0.396 ^a^ Island = 0.283 ^a^
Postoperative mobilisation, n (%)	852 (74.7)	744 (74.5)	115 (81.0)	Mainland = 0.99 (0.81 to 1.20) Island = 1.44 (0.94 to 2.28)	Mainland = 0.917 ^a^ Island = 0.101 ^a^
Acute LOS, median (IQR)	9 (6 to 13)	9 (6 to 14)	10 (8 to 16)	-	Mainland = 0.330 vs. non-transfer; 0.005 vs. island Island < 0.001 vs. non-transfer; 0.005 against mainland
Total LOS, median (IQR)	13 (8 to 30)	15 (8 to 32)	23 (12 to 17)	-	Mainland = 0.271 vs. non-transfer, <0.001 against island. Island <0.001 against non-transfer; <0.001 against mainland

n, number; %, percent; IQR, interquartile range; LOS, length of stay; OR, odds ratio; CI, confidence interval. ^a^ Unadjusted logistic regression. ^b^ Mann-Whitney U test.

**Table 5:** Sensitivity analysis of participants aged ≥80 and not admitted from home.

	Non-Transfer (n = 137)	Transfer (n = 137)	OR (95% CI)	*p*-Value
Surgery within 36 h, n (%)	114 (83.2)	92 (67.6)	0.42 (0.23 to 0.74)	0.003 ^a^
Time to surgery (hours), median (IQR)	19.4 (14.2 to 28.8)	26.4 (17.5 to 38.2)	-	<0.001 ^b^
30-day mortality, n (%)	14 (10.2)	21 (15.3)	1.59 (0.78 to 3.34)	0.208 ^a^
60-day mortality, n (%)	29 (21.2)	32 (23.4)	1.14 (0.65 to 2.03)	0.640 ^a^
Return to residence, n (%)	103 (75.2)	95 (69.3)	0.74 (0.44 to 1.27)	0.821 ^a^
Postoperative mobilisation, n (%)	79 (57.7)	71 (51.8)	0.78 (0.49 to 1.27)	0.332 ^a^
Acute LOS, median (IQR)	8 (6 to 11)	7 (5 to 10.3)	-	0.091 ^b^
Total LOS, median (IQR)	9 (6 to 14)	8 (5 to 17)	-	0.286 ^b^

n, number; %, percent; IQR, interquartile range; LOS, length of stay; OR, odds ratio; CI, confidence interval. ^a^ Unadjusted logistic regression. ^b^ Mann-Whitney U test.

## Data Availability

Requests for data included in the study should be made to Public Health Scotland. Code available for the project is available on request to the senior author.

## Appendix A

**Table A1:** Characteristics of included hip fracture patients and associations with transfer status before data imputation.

	Non-Transfer (n = 19,049)	Transfer (n = 1141)	Total (n = 20,190)
Sex, n (%)			
Female	13,446 (70.6)	799 (70.0)	14,245 (70.6)
Male	5603 (29.4)	342 (30.0)	5945 (29.4)
Missing	0 (0.0)	0 (0.0)	0 (0.0)
Age, n (%)			
50 to 54	300 (1.6)	30 (2.6)	330 (1.6)
55 to 59	519 (2.7)	38 (3.3)	557 (2.8)
60 to 64	752 (4.0)	54 (4.7)	806 (4.0)
65 to 69	1139 (6.0)	73 (6.4)	1212 (6.0)
70 to 74	2057 (10.8)	128 (11.2)	2185 (10.8)
75 to 79	2779 (14.6)	198 (17.4)	2977 (14.7)
80 to 84	3947 (20.7)	228 (20.0)	4175 (20.7)
85 to 89	4207 (22.1)	221 (19.4)	4428 (21.9)
90 to 94	2525 (13.3)	125 (11.0)	2650 (13.1)
95+	824 (4.3)	46 (4.0)	870 (4.3)
Missing	0 (0.0)	(0.0)	0 (0.0)
SIMD, n (%)			
1	1842 (9.7)	66 (5.8)	1908 (9.5)
2	1962 (10.3)	103 (9.0)	2065 (10.2)
3	1995 (10.5)	115 (10.1)	2110 (10.5)
4	1822 (9.6)	109 (9.6)	1931 (9.6)
5	1894 (9.9)	176 (15.4)	2070 (10.3)
6	1839 (9.7)	173 (15.2)	2012 (10.0)
7	1803 (9.5)	124 (10.9)	1927 (9.5)
8	1692 (8.9)	93 (8.2)	1785 (8.8)
9	1831 (9.6)	88 (7.7)	1919 (9.5)
10	1724 (9.1)	47 (4.1)	1771 (8.8)
Missing	645 (3.4)	47 (4.1)	692 (3.4)
Residence prior to admission, n (%)			
Home	15,317 (80.4)	962 (84.3)	16,279 (80.6)
Not home	3663 (19.2)	166 (14.5)	3829 (19.0)
Missing	69 (0.4)	13 (1.1)	82 (0.4)
4AT, n (%)			
Delirium unlikely	7453 (39.1)	574 (50.3)	8027 (39.8)
Possible Cognitive impairment	3823 (20.1)	214 (18.8)	4037 (20.0)
Possible delirium	3520 (18.5)	185 (16.2)	3705 (18.4)
Missing	4253 (22.3)	168 (14.7)	4421 (21.9)
ASA, n (%)			
1	296 (1.6)	33 (2.9)	329 (1.6)
2	3653 (19.1)	302 (26.5)	3955 (19.6)
3	9229 (48.5)	597 (52.3)	9826 (48.7)
4 and 5	2301 (12.1)	188 (16.5)	2489 (12.3)
Missing	3570 (18.7)	21 (1.8)	3591 (17.8)
Operation type, n (%)			
Fixation	8599 (45.1)	437 (38.3)	9036 (44.8)
Hemi arthroplasty	9139 (48.0)	606 (53.1)	9745 (48.3)
THR	1244 (6.5)	96 (8.4)	1340 (6.6)
Missing	67 (0.4)	2 (0.2)	69 (0.3)
